# Supplementary material for: Prediction of the Medicinal Mechanisms of Pinellia ternata Breitenbach, a Traditional Medicine for Gastrointestinal Motility Disorders, through Network Pharmacology
Source: Plants (Basel). 2022 May 19;11(10):1348. doi: 10.3390/plants11101348 (PMC9145079; doi:10.3390/plants11101348)
Supplement: Supplementary file 1 [file plants-11-01348-s001.zip › Supplementary Materials Table S1 Potential active compounds of Pinellia ternata Breitenbach.pdf]

Supplementary Materials Table S1

Potential active compounds of *Pinellia ternata Breitenbach.*

Molecule name	Structure	MW	OB(%)	Caco-2	DL
protocatechuic acid	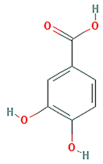	154.13	25.37	0.10	0.04
vanillic acid	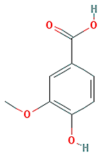	168.16	35.47	0.43	0.04
Neral	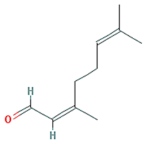	152.26	19.48	1.36	0.02
L-alpha-Palmitin	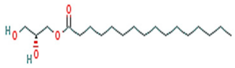	330.57	26.66	0.30	0.22
EIC	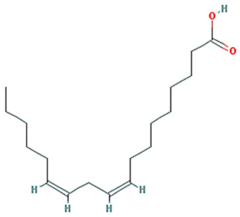	280.5	41.90	1.16	0.14
9-Oxononanoic acid	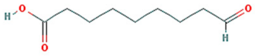	172.25	19.60	0.18	0.03
PENTADECYLIC ACID	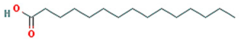	242.45	20.18	1.08	0.08

Molecule name	Structure	MW	OB(%)	Caco-2	DL
protocatechualdehyde	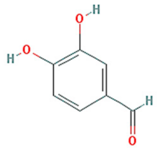	138.13	38.35	0.43	0.03
Ethyl icosanoate	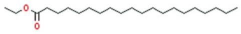	340.66	16.67	1.40	0.25
Furol	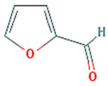	96.09	34.35	1.08	0.01
Crysophanol	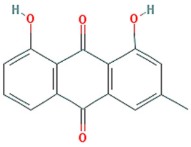	254.25	18.64	0.62	0.21
zoomaric acid	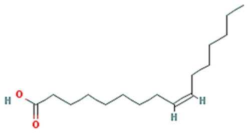	254.46	35.78	1.18	0.10
uracil	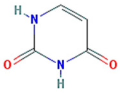	112.1	42.53	0.05	0.02
24-Ethylcholest-4-en-3-one	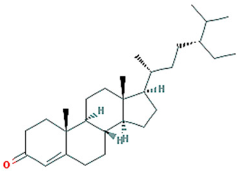	412.77	36.08	1.46	0.76

Molecule name	Structure	MW	OB(%)	Caco-2	DL
GUN	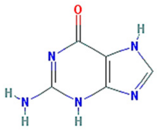	151.15	42.45	-0.17	0.04
adenine	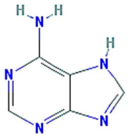	135.15	62.81	-0.30	0.03
Amide HPL	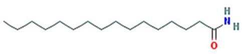	255.5	19.79	1.22	0.10
Methyl palmitelaidate	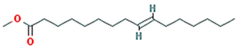	268.49	34.61	1.40	0.12
HX	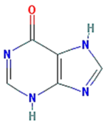	136.13	52.29	0.09	0.04
caffeic acid	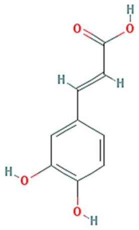	180.17	25.76	0.21	0.05
Barolub	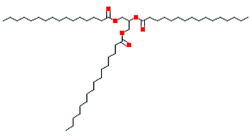	807.49	16.29	0.88	0.22

Molecule name	Structure	MW	OB(%)	Caco-2	DL
6-shogaol	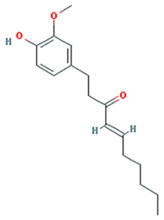	276.41	31.00	1.07	0.14
Cavidine	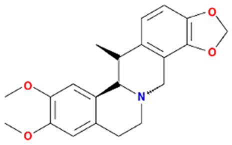	353.45	35.64	1.08	0.81
baicalein	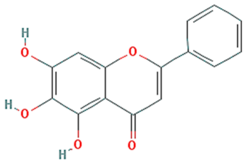	270.25	33.52	0.63	0.21
Baicalin	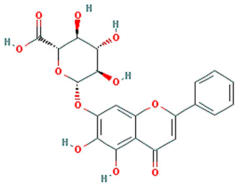	446.39	40.12	-0.85	0.75
succinic acid	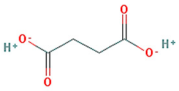	118.1	29.62	-0.44	0.01
Sitogluside	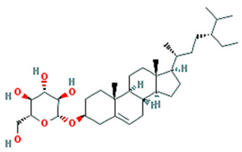	576.95	20.63	-0.14	0.62

Molecule name	Structure	MW	OB(%)	Caco-2	DL
beta-sitosterol	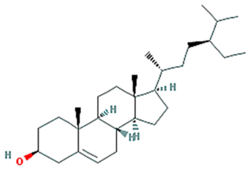	414.79	36.91	1.32	0.75
DL-Glucuronic acid	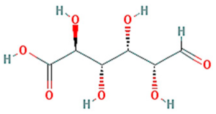	194.16	3.35	-1.91	0.04
gynesine	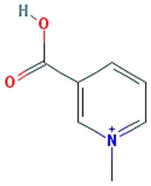	137.15	60.07	0.58	0.03
gamma-aminobutyric acid	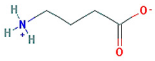	103.14	24.09	-0.26	0.01
FERULIC ACID (CIS)	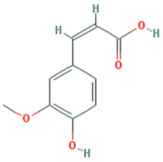	194.2	54.97	0.53	0.06
choline	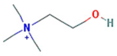	104.2	0.47	0.86	0.01
L-Serin	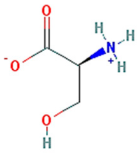	105.11	98.47	-1.12	0.01

Molecule name	Structure	MW	OB(%)	Caco-2	DL
cis-p-Coumarate	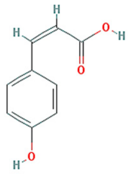	164.17	45.98	0.46	0.04
Threonin	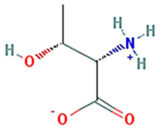	119.14	73.52	-0.87	0.01
Docosanoate	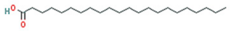	340.66	15.69	1.21	0.26
linolenic acid	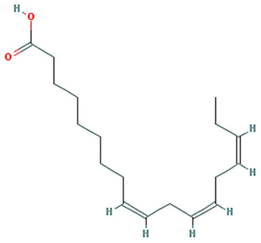	278.48	45.01	1.21	0.15
(2S)-2-amino-3-[(2R)-2-amino-3-hydroxy-3-oxopropyl]disulfanylpropanoic acid	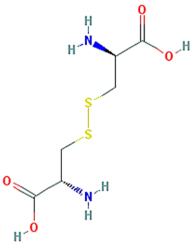	240.34	73.59	-1.23	0.05
Stigmasterol	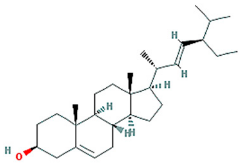	412.77	43.83	1.44	0.76

Molecule name	Structure	MW	OB(%)	Caco-2	DL
campesterol	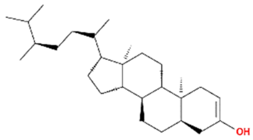	400.76	5.57	1.60	0.72
1-[(2R,3R,4S,5S)-3,4-dihydroxy-5-(hydroxymethyl)oxolan-2-yl]pyrimidine-2,4-dione	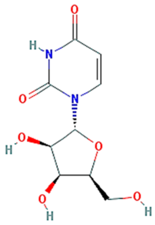	244.23	17.85	-1.19	0.11
Spantol	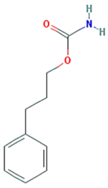	179.24	2.42	0.93	0.04
DAL	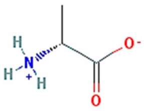	89.11	85.17	-0.30	0.01
anethole	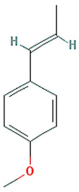	148.22	32.49	1.75	0.03
soya-cerebroside i	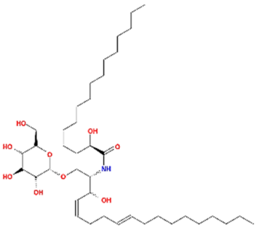	714.16	3.86	-1.09	0.37

Molecule name	Structure	MW	OB(%)	Caco-2	DL
soya-cerebroside i_qt	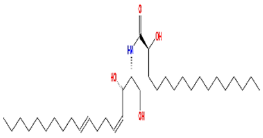	552	21.22	-0.02	0.51
GLY	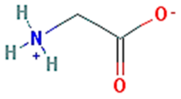	75.08	48.74	-0.56	0.00
gondoic acid	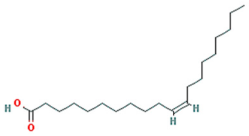	310.58	30.70	1.20	0.20
ANN	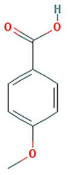	152.16	29.69	0.69	0.03
3,4,5-trihydroxybenzoic acid	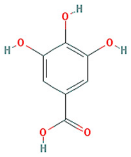	170.13	31.69	-0.09	0.04
coniferin	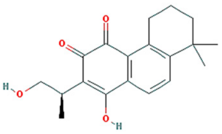	314.41	31.11	0.42	0.32

Molecule name	Structure	MW	OB(%)	Caco-2	DL
Gulutamine	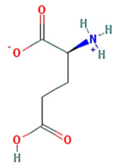	147.15	6.66	-1.05	0.02
L-Arginin	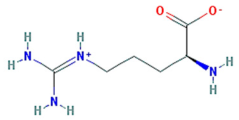	174.24	47.64	-0.49	0.03
Leucinum	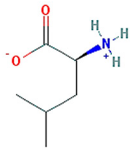	131.2	72.92	-0.05	0.01
L-Lysin	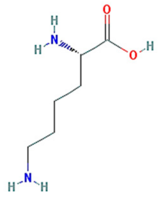	146.22	29.33	-0.66	0.02
DTY	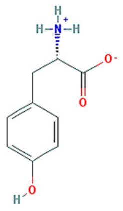	181.21	57.55	-0.10	0.05
hydroquinone	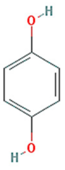	110.12	29.26	0.89	0.02

Molecule name	Structure	MW	OB(%)	Caco-2	DL
THM	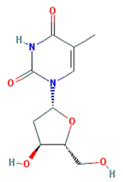	242.26	11.34	-0.89	0.11
ASI	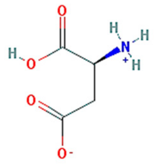	133.12	79.74	-1.02	0.02
Eciphin	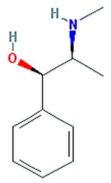	165.26	43.35	0.92	0.03
OMD	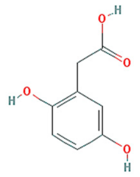	168.16	92.44	0.24	0.04
L-Valin	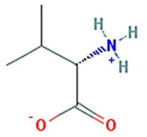	117.17	53.33	0.04	0.01
oleic acid	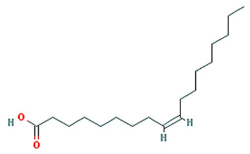	282.52	33.13	1.17	0.14

Molecule name	Structure	MW	OB(%)	Caco-2	DL
L-Ile	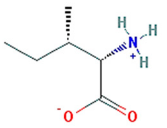	131.2	59.05	0.06	0.02
Norharman	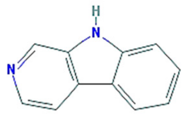	168.21	18.88	1.46	0.08
palmitic acid	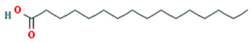	256.48	19.30	1.09	0.10
(+) isolariciresinol 9-o-β-d-glucopyranoside	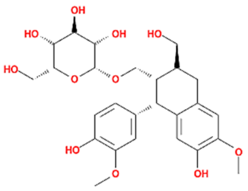	522.6	3.83	-1.17	0.84
isolariciresino	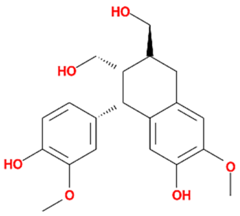	360.44	6.96	0.11	0.39
l-Pseudoephedrine	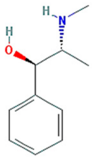	165.26	45.01	1.06	0.03

Molecule name	Structure	MW	OB(%)	Caco-2	DL
1,2,3,4,6-penta-o-galloyl-β-d-glucose	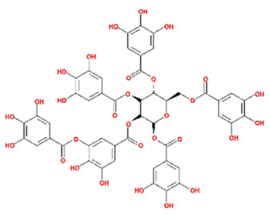	1092.83	3.01	-3.51	0.13
WLN: Q5Q	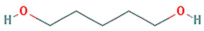	104.17	24.80	0.02	0.01
Octylene	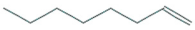	112.24	39.25	1.79	0.01
10,13-eicosadienoic	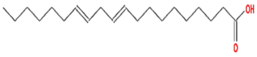	308.56	39.99	1.22	0.20
12,13-epoxy-9-hydroxynonadeca-7,10-dienoic acid	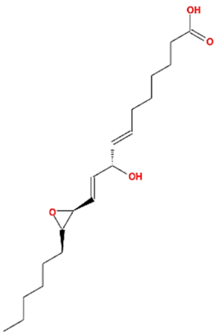	324.51	42.15	0.18	0.24
DUR	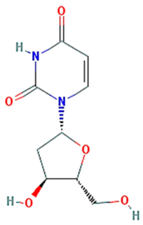	228.23	23.69	-0.97	0.09
Methylpyrazine	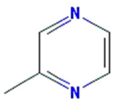	94.13	27.10	0.97	0.01

Molecule name	Structure	MW	OB(%)	Caco-2	DL
D-2-Aminobutyrate	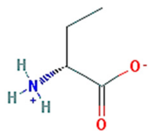	103.14	68.78	-0.16	0.01
3-methyleicosa	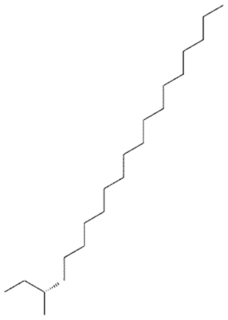	296.65	10.18	1.89	0.15
N-(5-methylisoxazol-3-yl)acetamide	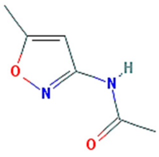	140.16	20.82	0.57	0.02
5,8-epidioxyergosta-6,22-dien-3-ol	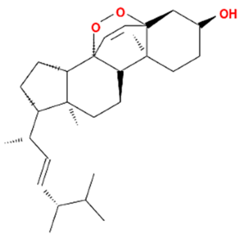	428.72	22.71	0.87	0.82
8-Octadecenoic acid	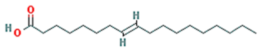	282.52	33.13	1.15	0.14
9-Heptadecanol	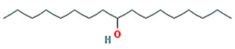	256.53	14.24	1.30	0.09

Molecule name	Structure	MW	OB(%)	Caco-2	DL
adenosine	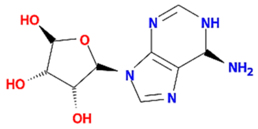	255.27	19.85	-1.49	0.16
heptadecanoic acid 2,3-dihydroxy-propyl ester	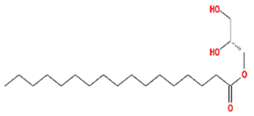	344.6	25.90	0.29	0.26
inosine	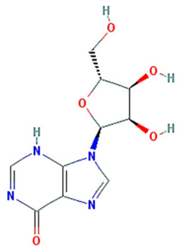	268.26	11.17	-1.12	0.18
6-Deoxy-gulose	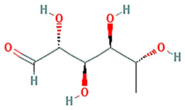	164.18	44.03	-1.11	0.03
(2R)-2-acetamidoglutaric acid	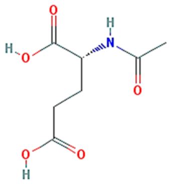	189.19	15.50	-0.76	0.04
pedatisectine a	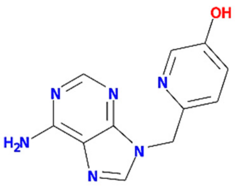	242.27	64.09	-0.30	0.16
pedatisectine f	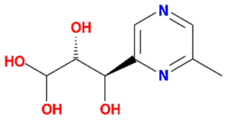	200.22	53.81	-0.85	0.06

Molecule name	Structure	MW	OB(%)	Caco-2	DL
Thy	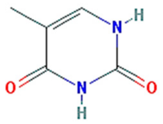	126.13	74.20	0.24	0.02
BVE	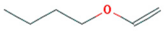	100.18	42.32	1.44	0.01
Cedrol	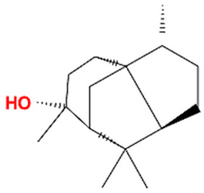	222.41	16.23	1.35	0.12
cyclo-(leu-tyr)	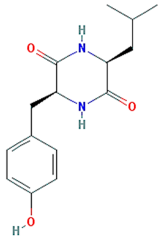	276.37	111.16	0.16	0.15
(3S,6S)-3-(benzyl)-6-(4-hydroxybenzyl)piperazine-2,5-quinone	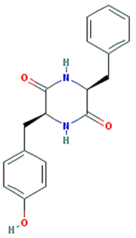	310.38	46.89	0.41	0.27
cyclo-(val-tyr)	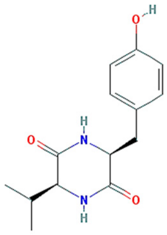	262.34	122.79	0.17	0.14

Molecule name	Structure	MW	OB(%)	Caco-2	DL
Cycloartenol	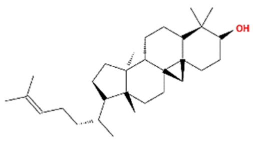	426.8	38.69	1.53	0.78
(5R)-5-hydroxy-1-(4-hydroxy-3-methoxyphenyl)tetradecan-3-one	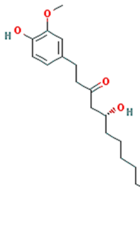	350.55	19.14	0.45	0.28
GR	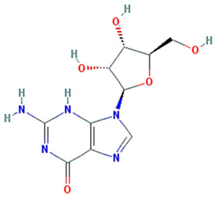	283.28	20.90	-1.22	0.21
2Z-hexadecenoic acid	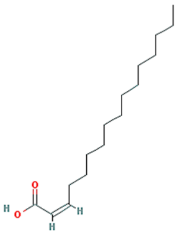	254.46	34.02	1.22	0.10
Ethyl linolelaidate	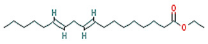	308.56	7.33	1.47	0.19
valeraldoxime	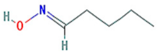	101.17	82.58	0.72	0.01
soya-cerebroside ii	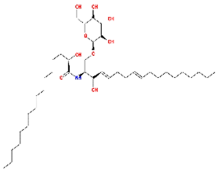	714.16	3.86	-1.30	0.35

Molecule name	Structure	MW	OB(%)	Caco-2	DL
soya-cerebroside ii_qt	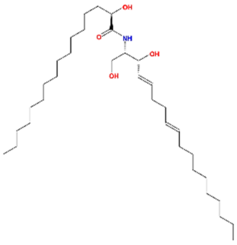	552	21.22	-0.16	0.49
beta-D-Ribofuranoside, xanthine-9	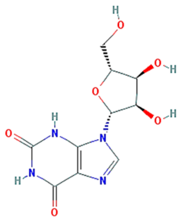	284.26	44.72	-1.21	0.21
MRY	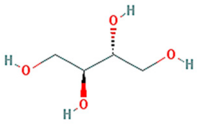	122.14	59.62	-1.30	0.01
WLN: VHR	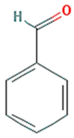	106.13	32.63	1.32	0.01
Istidina	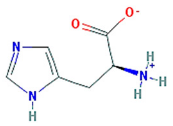	155.18	53.18	-0.25	0.03
HMF	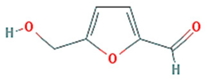	126.12	45.07	0.05	0.02
(-)-Citronellal	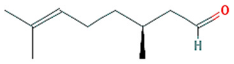	154.28	35.71	1.34	0.02

Molecule name	Structure	MW	OB(%)	Caco-2	DL
Glycerol palmitate	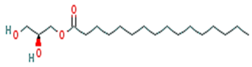	330.57	26.66	0.17	0.22
stearic acid	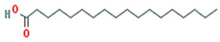	284.54	17.83	1.15	0.14
catechol	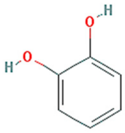	110.12	29.86	1.09	0.02
beta-elemene	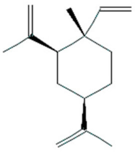	204.39	25.63	1.84	0.06
Mnk	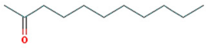	170.33	17.66	1.40	0.03
Ethylpalmitate	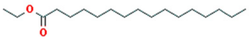	284.54	18.99	1.41	0.14
