# Supplementary material for: Prediction of the Medicinal Mechanisms of Pinellia ternata Breitenbach, a Traditional Medicine for Gastrointestinal Motility Disorders, through Network Pharmacology
Source: Plants (Basel). 2022 May 19;11(10):1348. doi: 10.3390/plants11101348 (PMC9145079; doi:10.3390/plants11101348)
Supplement: Supplementary file 1 [file plants-11-01348-s001.zip › Supplementary Materials Table S2 Target genes of Pinellia ternata Breitenbach.pdf]

Supplementary Materials Table S2

Target genes of *Pinellia ternata Breitenbach.*

Target Name	Gene name	UniprotID
1-aminocyclopropane-1-carboxylate deaminase	acdS	Q00740
2-amino-3-ketobutyrate coenzyme A ligase, mitochondrial	GCAT	O75600
2-hydroxy-6-oxo-7-methylocta-2,4-dienoate hydrolase	cumD	P96965
2-isopropylmalate synthase	leuA	P96420
3-hydroxy-3-methylglutaryl-coenzyme A reductase	mvaA	P13702
4-aminobutyrate aminotransferase, mitochondrial	ABAT	P80404
4-chlorobenzoyl CoA ligase		Q8GN86
5,6-dihydroxyindole-2-carboxylic acid oxidase	TYRP1	P17643
5-aminolevulinate synthase, erythroid-specific, mitochondrial	ALAS2	P22557
5-aminolevulinate synthase, nonspecific, mitochondrial	ALAS1	P13196
5-hydroxytryptamine 2A receptor	HTR2A	P28223
5-hydroxytryptamine 2C receptor	HTR2C	P28335
5-hydroxytryptamine 3 receptor	HTR3A	P46098
5-oxoprolinase	OPLAH	O14841
Acetolactate synthase, catabolic	budB	P27696
Acetylcholinesterase	ACHE	P22303
Acetyl-CoA acetyltransferase	phbA	P07097
Actin, cytoplasmic 1	ACTB	P60709
Acyl-CoA desaturase	SCD	O00767
Adenylosuccinate synthetase	purA	P0A7D4
Adenylosuccinate synthetase isozyme 1	ADSSL1	Q8N142
Alanine aminotransferase 1	GPT	P24298
Alanine aminotransferase 2	GPT2	Q8TD30
Alanine racemase	alr	P10724
Alanine--glyoxylate aminotransferase 2, mitochondrial	AGXT2	Q9BYV1
Alanine--glyoxylate aminotransferase 2-like 2	AGXT2L2	Q8IUZ5
Alanyl-tRNA synthetase, cytoplasmic	AARS	P49588
Aldehyde dehydrogenase, mitochondrial	ALDH2	P05091
Aldose reductase	AKR1B1	P15121
Alpha-1A adrenergic receptor	ADRA1A	P35348
Alpha-1B adrenergic receptor	ADRA1B	P35368
Alpha-1D adrenergic receptor	ADRA1D	P25100
Alpha-2A adrenergic receptor	ADRA2A	P08913

Target Name	Gene name	UniprotID
Alpha-2B adrenergic receptor	ADRA2B	P18089
Alpha-2C adrenergic receptor	ADRA2C	P18825
Alpha-ketoglutarate-dependent taurine dioxygenase	tauD	P37610
Amine oxidase [flavin-containing] A	MAOA	P21397
Amine oxidase [flavin-containing] B	MAOB	P27338
Androgen receptor	AR	P10275
Apolipoprotein D	APOD	P05090
Apoptosis regulator BAX	BAX	Q07812
Apoptosis regulator Bcl-2	BCL2	P10415
Arachidonate 12-lipoxygenase, 12S-type	ALOX12	P18054
Arachidonate 5-lipoxygenase	ALOX5	P09917
Arginase-2, mitochondrial	ARG2	P78540
Aromatic-amino-acid aminotransferase	tyrB	P95468
Aryl hydrocarbon receptor	AHR	P35869
Arylsulfatase	atsA	P51691
Asparagine synthetase [glutamine-hydrolyzing]	ASNS	P08243
Aspartate aminotransferase	aspC	P00509
Aspartate aminotransferase, cytoplasmic	GOT1	P17174
Aspartate aminotransferase, mitochondrial	GOT2	P00505
Aspartyl aminopeptidase	DNPEP	Q9ULA0
Bacillolysin	nprS	P43133
Bcl-2-like protein 1	BCL2L1	Q07817
Benzoylformate decarboxylase	mdlC	P20906
Beta-1 adrenergic receptor	ADRB1	P08588
Beta-2 adrenergic receptor	ADRB2	P07550
Beta-amylase	spoII	P36924
Beta-galactosidase	lacZ	P00722
Beta-lactamase	ampC	P00811
Beta-lactamase SHV-1	bla	P0AD64
Beta-lactamase SHV-1 precursor	bla	P0AD63
Beta-lactamase SHV-2 precursor	bla	P0A9Z7
Bifunctional polymyxin resistance protein arnA	arnA	P77398
Biphenyl-2,3-diol 1,2-dioxygenase	bphC	P47228
Brain-derived neurotrophic factor	BDNF	P23560
Branched-chain-amino-acid aminotransferase, cytosolic	BCAT1	P54687
Branched-chain-amino-acid aminotransferase, mitochondrial	BCAT2	O15382
Calcium-binding mitochondrial carrier protein Aralar1	SLC25A12	O75746

Target Name	Gene name	UniprotID
Calcium-binding mitochondrial carrier protein Aralar2	SLC25A13	Q9UJS0
Calcium-transporting ATPase type 2C member 1	ATP2C1	P98194
Calmodulin	CALM1	P62158
cAMP and cAMP-inhibited cGMP 3',5'-cyclic phosphodiesterase 10A	PDE10A	Q9Y233
cAMP-dependent protein kinase catalytic subunit alpha	PRKACA	P17612
cAMP-dependent protein kinase inhibitor alpha	PKIA	P61925
Carbonic anhydrase 2	CA2	P00918
Carnitine O-palmitoyltransferase 2, mitochondrial	CPT2	P23786
Caspase-3	CASP3	P42574
Caspase-8	CASP8	Q14790
Caspase-9	CASP9	P55211
Catechol O-methyltransferase	COMT	P21964
Catenin beta-1	CTNNB1	P35222
Cathepsin D	CTSD	P07339
Cationic amino acid transporter 4	SLC7A4	O43246
Cbp/p300-interacting transactivator 1	CITED1	Q99966
Cell division control protein 2 homolog	CDK1	P06493
Cell division protein kinase 2	CDK2	P24941
Cell-death-related nuclease 7	crn-7	P34387
Cellular tumor antigen p53	TP53	P04637
cGMP-inhibited 3',5'-cyclic phosphodiesterase A	PDE3A	Q14432
Cholecystokinin	CCK	P06307
Cholesteryl ester transfer protein	CETP	P11597
Choline/ethanolamine kinase [Includes: Choline kinase beta	CHKB	Q9Y259
Choline-phosphate cytidylyltransferase A	PCYT1A	P49585
Cholinesterase	BCHE	P06276
Chymotrypsin-like elastase family member 1	CELA1	Q9UNI1
Chymotrypsinogen B	CTRB1	P17538
Clavaminate synthase 1	cs1	Q05581
Coagulation factor VII	F7	P08709
Coagulation factor X	F10	P00742
Coagulation factor XIII A chain	F13A1	P00488
C-reactive protein	CRP	P02741
Creatine kinase M-type	CKM	P06732
Cyclin-A2	CCNA2	P20248
Cyclin-dependent kinase inhibitor 1	CDKN1A	P38936

Target Name	Gene name	UniprotID
Cystathionine gamma-lyase	CTH	P32929
Cysteine desulfurase, mitochondrial	NFS1	Q9Y697
Cytochrome c	CYCS	P99999
Cytochrome P450 1A1	CYP1A1	P04798
Cytochrome P450 3A43	CYP3A43	Q9HB55
Cytochrome P450-cam	camC	P00183
D(1A) dopamine receptor	DRD1	P21728
D-alanyl-D-alanine carboxypeptidase		P15555
Deacetoxycephalosporin C synthetase	cefE	P18548
Delta-type opioid receptor	OPRD1	P41143
Dihydroorotase	pyrC	P05020
Dihydroxyacetone kinase	dhaK	P45510
Dipeptidyl peptidase 4	DPP4	P27487
DNA damage-inducible transcript 3 protein	DDIT3	P35638
DNA polymerase	UL30	P04293
DNA topoisomerase 2-alpha	TOP2A	P11388
Ectonucleotide pyrophosphatase/phosphodiesterase family member 7	ENPP7	Q6UWV6
Egl nine homolog 1	EGLN1	Q9GZT9
Endoglucanase G	celCCG	P37700
Endothelin-1	EDN1	P05305
Estrogen receptor	ESR1	P03372
Estrogen receptor beta	ESR2	Q92731
Eukaryotic translation initiation factor 6	EIF6	P56537
Fatty acid synthase	FASN	P49327
Fatty acid-binding protein, epidermal	FABP5	Q01469
Fatty acid-binding protein, liver	FABP1	P07148
Ferredoxin-dependent glutamate synthase 2	gltS	P55038
Ferrichrome-iron receptor	fhuA	P06971
Formate acetyltransferase 1	pflB	P09373
Formate dehydrogenase H	fdhF	P07658
Fos-related antigen 1	FOSL1	P15407
Fos-related antigen 2	FOSL2	P15408
Fumarate reductase flavoprotein subunit	SO_0970	P83223
G2/mitotic-specific cyclin-B1	CCNB1	P14635
Gag-Pol polyprotein	gag-pol	P12497
Gamma-aminobutyraldehyde dehydrogenase	prr	P77674

Target Name	Gene name	UniprotID
Gamma-aminobutyric-acid receptor subunit alpha-1	GABRA1	P14867
Gamma-aminobutyric-acid receptor subunit alpha-2	GABRA2	P47869
Gamma-aminobutyric-acid receptor subunit alpha-3	GABRA3	P34903
Gamma-aminobutyric-acid receptor subunit alpha-5	GABRA5	P31644
Gamma-aminobutyric-acid receptor subunit alpha-6	GABRA6	Q16445
Gephyrin	GPHN	Q9NQX3
Glial fibrillary acidic protein	GFAP	P14136
Glucagon	GCG	P01275
Glucarate dehydratase	gudD	P0AES2
Glucose-6-phosphate isomerase	GPI	P06744
Glucose--fructose oxidoreductase	gfo	Q07982
Glutamate [NMDA] receptor subunit 3A	GRIN3A	Q8TCU5
Glutamate [NMDA] receptor subunit epsilon-1	GRIN2A	Q12879
Glutamate [NMDA] receptor subunit epsilon-2	GRIN2B	Q13224
Glutamate [NMDA] receptor subunit epsilon-3	GRIN2C	Q14957
Glutamate [NMDA] receptor subunit zeta-1	GRIN1	Q05586
Glutamate dehydrogenase 1, mitochondrial	GLUD1	P00367
Glutamate dehydrogenase 2, mitochondrial	GLUD1	P00367
Glutamate receptor 1	GRIA1	P42261
Glutamate receptor 2	GRIA2	P42262
Glutamate receptor, ionotropic kainate 2	GRIK2	Q13002
Glutamyl aminopeptidase	ENPEP	Q07075
Glycine amidinotransferase, mitochondrial	GATM	P50440
Glycine N-acyltransferase	GLYAT	Q6IB77
Glycine N-acyltransferase-like protein 1	GLYATL1	Q969I3
Glycine N-methyltransferase	GNMT	Q14749
Glycine oxidase	thiO	O31616
Glycine receptor subunit alpha-1	GLRA1	P23415
Glycine receptor subunit alpha-2	GLRA2	P23416
Glycine receptor subunit alpha-3	GLRA3	O75311
Glycogen phosphorylase, liver form	PYGL	P06737
Glycogen phosphorylase, muscle form	PYGM	P11217
Glycogen synthase 1	glgA1	P0A3F3
Glycogen synthase kinase-3 beta	GSK3B	P49841
Glycyl-tRNA synthetase	GARS	P41250
Group IIE secretory phospholipase A2	PLA2G2E	Q9NZK7
Growth-inhibiting protein 18	GIG18	Q2TU84

Target Name	Gene name	UniprotID
Haloalkane dehalogenase	linB	P51698
Heat shock protein HSP 90-alpha	HSP90AA1	P07900
Hepatocyte nuclear factor 1-alpha	HNF1A	P20823
Hepatocyte nuclear factor 4-alpha	HNF4A	P41235
High-affinity cationic amino acid transporter 1	SLC7A1	P30825
Histidinol dehydrogenase	hisD	P06988
Hydroxylamine reductase	hcp	Q01770
Hypothetical protein DKFZp686P09201	DKFZp686P09201	Q5HYG8
Hypoxia-inducible factor 1-alpha	HIF1A	Q16665
Ig gamma-1 chain C region	IGHG1	P01857
Inhibitor of nuclear factor kappa-B kinase subunit beta	IKBKB	O14920
Insulin	INS	P01308
Insulin-like growth factor II	IGF2	P01344
Interleukin-10	IL10	P22301
Interleukin-2	IL2	P60568
Interleukin-6	IL6	P05231
Kynureninase	KYNU	Q16719
Lactotransferrin	LTF	P02788
Leukotriene A-4 hydrolase	LTA4H	P09960
Lipoprotein lipase	LPL	P06858
L-lactate dehydrogenase A chain	LDHA	P00338
L-lactate dehydrogenase B chain	LDHB	P07195
L-serine dehydratase	SDS	P20132
Lysozyme	E	P00720
Macrophage metalloelastase	MMP12	P39900
Malate dehydrogenase	mdh	P80040
Malonamidase E2		Q9ZIV5
Maltase-glucoamylase, intestinal	MGAM	O43451
Matrix metalloproteinase-9	MMP9	P14780
Methionine synthase	metH	P13009
Methionyl-tRNA synthetase	metG	P00959
Methylmalonyl-CoA carboxyltransferase 5S subunit		Q70AC7
Microsomal glutathione S-transferase 1	MGST1	P10620
Microtubule-associated protein 2	MAP2	P11137
Migration-inducing protein 4	ALAS1	Q5JAM2
Mineralocorticoid receptor	NR3C2	P08235
Mitochondrial aspartate-glutamate carrier protein	SLC25A13	Q546F9

Target Name	Gene name	UniprotID
Mitochondrial dicarboxylate carrier	SLC25A10	Q9UBX3
Mitochondrial uncoupling protein 2	UCP2	P55851
Mitochondrial uncoupling protein 3	UCP3	P55916
Mitogen-activated protein kinase 14	MAPK14	Q16539
Monomeric sarcosine oxidase	soxA	P40859
M-phase inducer phosphatase 2	CDC25B	P30305
Muscarinic acetylcholine receptor M1	CHRM1	P11229
Muscarinic acetylcholine receptor M2	CHRM2	P08172
Muscarinic acetylcholine receptor M3	CHRM3	P20309
Muscarinic acetylcholine receptor M4	CHRM4	P08173
Muscarinic acetylcholine receptor M5	CHRM5	P08912
Mu-type opioid receptor	OPRM1	P35372
Myc proto-oncogene protein	MYC	P01106
Myeloperoxidase	MPO	P05164
N-acetylglutamate synthase, mitochondrial	NAGS	Q8N159
NAD-dependent malic enzyme, mitochondrial	ME2	P23368
NADP-dependent malic enzyme	ME1	P48163
NADP-dependent malic enzyme, mitochondrial	ME3	Q16798
NADPH oxidase 5	NOX5	Q96PH1
Neuromodulin	GAP43	P17677
Neuronal acetylcholine receptor subunit alpha-2	CHRNA2	Q15822
Neuronal acetylcholine receptor subunit alpha-7	CHRNA7	P36544
Neutrophil collagenase	MMP8	P22894
NF-kappa-B inhibitor alpha	NFKBIA	P25963
Nicotinate-nucleotide--dimethylbenzimidazole phosphoribosyltransferase	cobT	Q05603
Nitric-oxide synthase, endothelial	NOS3	P29474
Nitric-oxide synthase, brain	NOS1	P29475
Nuclear factor of activated T-cells, cytoplasmic 1	NFATC1	O95644
Nuclear receptor coactivator 1	NCOA1	Q15788
Nuclear receptor coactivator 2	NCOA2	Q15596
Oligopeptide ABC transporter, periplasmic oligopeptide-binding protein	TM_1223	Q9X0V0
Ornithine aminotransferase, mitochondrial	OAT	P04181
Ornithine carbamoyltransferase, mitochondrial	OTC	P00480
Ornithine decarboxylase	ODC1	P11926
Oxidoreductase	HSD17B6	O14756

Target Name	Gene name	UniprotID
Pancreas/duodenum homeobox protein 1	PDX1	P52945
Pancreatic alpha-amylase	AMY2A	P04746
Parathion hydrolase	opd	P0A433
Penicillin G acylase	pac	P06875
Peptide YY	PYY	P10082
Peptidyl-glycine alpha-amidating monooxygenase	PAM	P19021
Peroxidase/catalase T	katG	Q08129
Peroxisomal sarcosine oxidase	PIPOX	Q9P0Z9
Peroxisome proliferator-activated receptor alpha	PPARA	Q07869
Peroxisome proliferator-activated receptor delta	PPARD	Q03181
Peroxisome proliferator-activated receptor gamma	PPARG	P37231
Phosphatidylinositol-3,4,5-trisphosphate 3-phosphatase and dual-specificity protein phosphatase PTEN	PTEN	P60484
Phosphatidylinositol-4,5-bisphosphate 3-kinase catalytic subunit gamma isoform	PIK3CG	P48736
Phospholipase A2	PLA2G1B	P04054
Phosphonoacetaldehyde hydrolase	phnX	O31156
Phosphoribosylformylglycinamidine synthase	PFAS	O15067
Phosphotriesterase	opdA	Q93LD7
Plasminogen	PLG	P00747
Plasminogen activator inhibitor 1	SERPINE1	P05121
Potassium voltage-gated channel subfamily H member 2	KCNH2	Q12809
Probable alanyl-tRNA synthetase, mitochondrial	AARS2	Q5JTZ9
Probable pyruvate-flavodoxin oxidoreductase	ydbK	P52647
Progesterone receptor	PGR	P06401
Proliferating cell nuclear antigen	PCNA	P12004
Prolyl 3-hydroxylase 1	LEPRE1	Q32P28
Prolyl 3-hydroxylase 3	LEPREL2	Q8IVL6
Prolyl 4-hydroxylase subunit alpha-2	P4HA2	O15460
Prostaglandin G/H synthase 1	PTGS1	P23219
Prostaglandin G/H synthase 2	PTGS2	P35354
Protein CBFA2T1	RUNX1T1	Q06455
Protein kinase C alpha type	PRKCA	P17252
Protein kinase C beta type	PRKCB	P05771
Protein kinase C gamma type	PRKCG	P05129
Protein kinase C zeta type	PRKCZ	Q05513
Prothrombin	F2	P00734

Target Name	Gene name	UniprotID
Proton-coupled amino acid transporter 1	SLC36A1	Q7Z2H8
Proto-oncogene c-Fos	FOS	P01100
Proto-oncogene serine/threonine-protein kinase Pim-1	PIM1	P11309
Proto-oncogene tyrosine-protein kinase Src	SRC	P12931
P-selectin	SELP	P16109
Purine nucleoside phosphorylase	PNP	P00491
Purine nucleoside phosphorylase deoD-type	deoD	P0ABP8
Putative adenosylhomocysteinase 2	LOC107711996	A0A67314K8
Putative beta-glucuronidase-like protein SMA3	GUSBP1	Q15486
Pyruvate carboxylase, mitochondrial	PC	P11498
Pyruvate dehydrogenase [cytochrome]	poxB	P07003
Pyruvate dehydrogenase E1 component subunit beta, mitochondrial	PDHB	P11177
Pyruvate kinase isozymes M1/M2	PKM2	P14618
Pyruvate-ferredoxin oxidoreductase	por	P94692
RAC-alpha serine/threonine-protein kinase	AKT1	P31749
Ras-related C3 botulinum toxin substrate 1	RAC1	P63000
Receptor tyrosine-protein kinase erbB-2	ERBB2	P04626
Renin	REN	P00797
Retinoblastoma-associated protein	RB1	P06400
Retinoic acid receptor RXR-alpha	RXRA	P19793
Retinoic acid receptor RXR-gamma	RXRG	P48443
Retinol-binding protein 2	RBP2	P50120
Rhodopsin	RHO	P08100
S-adenosylmethionine decarboxylase proenzyme	AMD1	P17707
S-adenosylmethionine synthetase isoform type-1	MAT1A	Q00266
S-adenosylmethionine synthetase isoform type-2	MAT2A	P31153
Sensor protein fixL	fixL	P23222
Serine hydroxymethyltransferase, cytosolic	SHMT1	P34896
Serine hydroxymethyltransferase, mitochondrial	SHMT2	P34897
Serine/threonine-protein kinase Chk1	CHEK1	O14757
Serine--pyruvate aminotransferase	AGXT	P21549
Serum albumin		Q56G89
Serum paraoxonase/arylesterase 1	PON1	P27169
Seryl-tRNA synthetase, cytoplasmic	SARS	P49591
SHMT2 protein	SHMT2	Q5BJF5
Sigma factor sigB regulation protein rsbQ	rsbQ	O07015

Target Name	Gene name	UniprotID
Siroheme synthase	cysG	P25924
Sodium- and chloride-dependent GABA transporter 1	SLC6A1	P30531
Sodium- and chloride-dependent glycine transporter 1	SLC6A9	P48067
Sodium channel protein type 5 subunit alpha	SCN5A	Q14524
Sodium-dependent dopamine transporter	SLC6A3	Q01959
Sodium-dependent noradrenaline transporter	SLC6A2	P23975
Sodium-dependent serotonin transporter	SLC6A4	P31645
Solute carrier family 2, facilitated glucose transporter member 2	SLC2A2	P11168
Solute carrier family 22 member 5	SLC22A5	O76082
Sterol O-acyltransferase 1	SOAT1	P35610
Succinate dehydrogenase [ubiquinone] flavoprotein subunit, mitochondrial	SDHA	P31040
Succinate dehydrogenase flavoprotein subunit	sdhA	P0AC41
Succinate semialdehyde dehydrogenase, mitochondrial	ALDH5A1	P51649
Succinyl-CoA ligase [ADP-forming] beta-chain, mitochondrial	SUCLA2	Q9P2R7
Succinyl-CoA:3-ketoacid-coenzyme A transferase 2, mitochondrial	OXCT2	Q9BYC2
Superoxide dismutase [Cu-Zn]	SOD1	P00441
Telomerase protein component 1	TEP1	Q99973
Thermolysin	npr	P00800
Thioredoxin reductase 1, cytoplasmic	TXNRD1	Q16881
Threonyl-tRNA synthetase, cytoplasmic	TARS	P26639
Transcription factor AP-1	JUN	P05412
Transcription factor p65	RELA	Q04206
Transcription factor Sp1	SP1	P08047
Transcriptional activator Myb	MYB	P10242
Transforming growth factor beta-1	B9D2	F1PMQ2
Transforming protein RhoA	RHOA	P61586
Transient receptor potential cation channel subfamily V member 1	TRPV1	Q8NER1
Transitional endoplasmic reticulum ATPase	VCP	P55072
Triosephosphate isomerase	TPI1	P60174
Tripartite motif-containing protein 26	TRIM26	Q12899
Trypsin-1	PRSS1	P07477
Trypsin-3	PRSS3	P35030
Tudor domain-containing protein 7	TDRD7	Q8NHU6

Target Name	Gene name	UniprotID
Tumor necrosis factor	TNF	P01375
Tumor necrosis factor ligand superfamily member 6	FASLG	P48023
Tyrosine-protein kinase BTK	BTK	Q06187
Tyrosine-protein phosphatase non-receptor type 1	PTPN1	P18031
Tyrosyl-tRNA synthetase, mitochondrial	YARS2	Q9Y2Z4
UDP-glucose 4-epimerase	GALE	Q14376
Urease alpha subunit	ureC	P18314
Urokinase-type plasminogen activator	PLAU	P00749
V-type proton ATPase catalytic subunit A	ATP6V1A	P38606
Xanthine dehydrogenase/oxidase	XDH	P47989
Y+L amino acid transporter 1	SLC7A7	Q9UM01
