# Supplementary material for: Prediction of the Medicinal Mechanisms of Pinellia ternata Breitenbach, a Traditional Medicine for Gastrointestinal Motility Disorders, through Network Pharmacology
Source: Plants (Basel). 2022 May 19;11(10):1348. doi: 10.3390/plants11101348 (PMC9145079; doi:10.3390/plants11101348)
Supplement: Supplementary file 1 [file plants-11-01348-s001.zip › Supplementary Materials Table S3 100 gastrointestinal motility disorder related genes.pdf]

Supplementary Materials Table S3

One hundred gastrointestinal motility disorder–related genes.

Gene name	Protein name
FOS	FBJ murine osteosarcoma viral oncogene homolog
PYY	Peptide tyrosine tyrosine
GUCA2A	Guanylate cyclase activator 2A (guanylin)
HTR7	5-hydroxytryptamine (serotonin) receptor 7, adenylate cyclase-coupled
MPO	Myeloperoxidase
GRP	Gastrin-releasing peptide
HTR2B	5-hydroxytryptamine (serotonin) receptor 2B, G protein-coupled
GDNF	Glial cell line-derived neurotrophic factor
MYH11	Myosin heavy chain, smooth muscle isoform
GPR55	G protein-coupled receptor 55
SLC10A2	Solute carrier family 10 (sodium/bile acid cotransporter), member 2
CFTR	Cystic fibrosis transmembrane conductance regulator (ATP-binding cassette sub-family C, member 7)
VIP	Vasoactive intestinal peptide
GPR119	Glucose-dependent insulinotropic receptor
CXCL8	Monocyte-derived neutrophil chemotactic factor
CRHR2	Corticotropin releasing hormone receptor 2
CCK	Cholecystokinin
TACR1	Tachykinin receptor 1
NPY	Pro-neuropeptide Y
PDZD3	Natrium-phosphate cotransporter IIa C-terminal-associated protein 2
GPBAR1	G protein-coupled bile acid receptor 1
POMC	Corticotropin-lipotropin
TRPV1	Transient receptor potential cation channel, subfamily V, member 1
IL10	Cytokine synthesis inhibitory factor
HOXB5	Homeobox protein HHO.C10
ATP4A	ATPase, H+/K+ exchanging, alpha polypeptide
GAPDH	Glyceraldehyde-3-phosphate dehydrogenase
MYLK	Myosin light chain kinase, smooth muscle
ELAVL4	ELAV like neuron-specific RNA binding protein 4
PHOX2B	Paired mesoderm homeobox protein 2B
ANO1	Discovered on gastrointestinal stromal tumors protein 1
KLB	Klotho beta-like protein
TACR2	Neurokinin A receptor
CYP7A1	Cytochrome P450, family 7, subfamily A, polypeptide 1

Gene name	Protein name
GHRL	Growth hormone-releasing peptide
SNCA	Synuclein, alpha (non A4 component of amyloid precursor)
CLDN2	Claudin 2
PTGS2	Prostaglandin-endoperoxide synthase 2 (prostaglandin G/H synthase and cyclooxygenase)
GHSR	Growth hormone secretagogue receptor type 1
CALB2	29 kDa calbindin
CCL28	Mucosae-associated epithelial chemokine
NR1H4	Nuclear receptor subfamily 1, group H, member 4
CRH	Corticotropin releasing hormone
SLC18A3	Solute carrier family 18 (vesicular acetylcholine transporter), member 3
SCT	Secretin
PPP1R14A	Protein phosphatase 1, regulatory (inhibitor) subunit 14A
KITLG	Mast cell growth factor
KIT	V-kit Hardy-Zuckerman 4 feline sarcoma viral oncogene homolog
GCG	Glucagon
TAC1	Tachykinin, precursor 1
TRPA1	Transient receptor potential cation channel, subfamily A, member 1
EDN3	Preproendothelin-3
OPRM1	Mu-type opioid receptor
NPSR1	G-protein coupled receptor for asthma susceptibility
FFAR3	G-protein coupled receptor 41
CCKAR	Cholecystokinin receptor type A
GUCY2C	Guanylate cyclase 2C (heat stable enterotoxin receptor)
HRH2	Histamine H2 receptor
S100B	S100 calcium binding protein B
ATP12A	ATPase, H+/K+ transporting, nongastric, alpha polypeptide
ACHE	Acetylcholinesterase (Yt blood group)
RET	Proto-oncogene tyrosine-protein kinase receptor Ret
MBOAT4	Membrane-bound O-acyltransferase domain-containing protein 4
UCHL1	Ubiquitin carboxyl-terminal esterase L1 (ubiquitin thiolesterase)
GAL	galanin/GMAP prepropeptide
IL1B	Interleukin 1, beta
TNF	Tumor necrosis factor ligand superfamily member 2
CRHR1	Corticotropin releasing hormone receptor 1
TYMP	Platelet-derived endothelial cell growth factor
GAST	Gastrin
PPY	Pancreatic polypeptide
ACTB	Actin, cytoplasmic 1

Gene name	Protein name
TLR4	Toll-like receptor 4
SOX10	SRY (sex determining region Y)-box 10
SCN5A	Sodium channel, voltage-gated, type V, alpha subunit
TH	Tyrosine 3-monooxygenase
CHAT	Choline O-acetyltransferase
ELAVL3	Paraneoplastic cerebellar degeneration-associated antigen
INS	Insulin
SLC6A4	Solute carrier family 6 (neurotransmitter transporter), member 4
FGF19	Fibroblast growth factor 19
CALCA	Calcitonin-related polypeptide alpha
CLCN2	Chloride channel, voltage-sensitive 2
NPR3	Atrial natriuretic peptide clearance receptor
MLNR	G-protein coupled receptor 38
TPH1	Tryptophan hydroxylase 1
OCLN	Occludin
CRP	C-reactive protein, pentraxin-related
ALB	Serum albumin
EDNRB	Endothelin receptor non-selective type
FFAR2	G-protein coupled receptor 43
IL6	B-cell stimulatory factor 2
MLN	Promotilin
NTS	Neurotensin/neuromedin N
NOS1	Peptidyl-cysteine S-nitrosylase NOS1
SST	Growth hormone release-inhibiting factor
HTR4	5-hydroxytryptamine (serotonin) receptor 4, G protein-coupled
RNPC3	U11/U12 small nuclear ribonucleoprotein 65 kDa protein
GIP	Glucose-dependent insulinotropic polypeptide
NGF	Nerve growth factor (beta polypeptide)
